# Toxic effect of carbon monoxide and 41 inflammatory cytokines: A bidirectional 2-sample Mendelian randomization study

**DOI:** 10.1097/MD.0000000000044596

**Published:** 2025-09-19

**Authors:** Huan Yang, Manli Sun, Lijie Zhao, Jingqi Ruan, Shichang Li, Yang Xu, Mingjing Duan, Yulei Bi

**Affiliations:** a Poisoning Department, Heilongjiang Province Poisoning Rescue and Treatment Center, The Second Hospital of Heilongjiang Province, Harbin, China; b Chinese Medicine Internal Department, Yuejin Community Health Service Center, Harbin, China.

**Keywords:** carbon monoxide (CO) poisoning, IL-13, IL-10, IL-1β, inflammatory cytokines, Mendelian randomization (MR)

## Abstract

Carbon monoxide (CO) poisoning causes an important public health problem, tens of thousands of deaths reported on a global scale each year. Previous research has identified various inflammatory cytokines as key players in the inflammatory response associated with CO poisoning. Mendelian randomization (MR) was utilized to rate the causation between CO poisoning and 41 inflammatory cytokines. Our research applied the MR method to analyze the data from 41 inflammatory cytokines in 8293 Finnish individuals, alongside the toxic effect of CO in a genome-wide association study summary containing 2,17,123 individuals. The inverse-variance weighting approach was adopted for the bidirectional MR analysis to explore these relationships, supplemented by multiple MR methods to ensure robustness. Two analytical methods were utilized to assess the assumptions, including the MR-Egger intercept test and the MR-PRESSO test. Our results of the 2-sample MR analysis revealed that IL-10, IL-1β and IL-13 had causal effects on CO toxicity (IL-10: β: 0.52; 95% CI: 0.02, 1.03; *P *= 4.19E−02; IL-1β: β: −1.23; 95% CI: −2.27, −0.20; *P* = 1.91E−02; IL-13: β: 0.54; 95% CI: 0.24, 0.85; *P *= 4.77E−04), while CO toxicity showed no significant causal effect on any of the 41 inflammatory cytokines. Our study suggests that IL-10, IL-1β, and IL-13 may be important players in immune response regulation, impacting CO poisoning prognosis. Our research offers fresh perspectives on the disease mechanisms underlying CO poisoning and pinpoints potential therapeutic targets for intervention.

## 1. Introduction

Carbon monoxide (CO) poisoning has become an accurate and prevalent public health issue, resulting in approximately 30,000 of deaths annually worldwide^[1]^ and resulting in lasting neurological impairments that profoundly affect patients' quality of life and impose considerable financial strains on their families.^[2]^ Currently, hyperbaric oxygen therapy is utilized to impede the growth of neurological sequelae. On the contrary, it has limited efficacy in improving long-term outcomes and preventing delayed encephalopathy.^[3]^ Therefore, understanding the pathogenic mechanisms of CO poisoning is of critical clinical importance.

CO can function as a chemical asphyxiant at high ambient concentrations. This is primarily attributed to CO's exceptionally high binding affinity for hemoglobin (Hb), which surpasses oxygen's by approximately 250 times.^[4]^ The partial binding of CO to the oxygen-binding site on the Hb suppresses the release of oxygen from other heme groups. The resultant accumulation of CO-Hb induces hypoxemia and compromises the delivery of oxygen to tissues, potentially leading to significant morbidity or fatality. CO can also cause harm through mechanisms including its binding to cytochrome c oxidase, triggering inflammatory responses, oxidative stress, and cell necrosis and apoptosis.^[5]^ Beyond the classical hypoxia-driven pathology, emerging evidence suggests that inflammatory responses play critical roles in the pathophysiology and clinical outcomes of CO poisoning, potentially influencing both acute symptoms and chronic complications.^[6]^ The inflammation triggered by CO involves activating multiple signaling pathways, notably the nuclear factor kappa B (NF-κB) pathway, the mitogen-activated protein kinase (MAPK) pathway, and the mTOR signaling pathway. Among them, NF-κB is an important players in modulating the expressions of pro-inflammatory cytokines and adhesion molecules, which become key players in motivating immune cells to the injured part. Studies have shown that CO exposure is capable of triggering the phosphorylation and subsequently activating NF-κB, resulting in high tumor necrosis factor-alpha and interleukin-6 (IL-6).^[7]^ Concurrently, the MAPK pathway, particularly the p38 MAPK pathway, is activated as a response to CO, resulting in improved inflammatory signaling and cellular stress responses.^[8]^ Additionally, recent research emphasizes the function of the mTOR pathway in mediating autophagy and inflammation during CO poisoning, suggesting that interventions targeting this pathway may provide therapeutic benefits.^[9]^

Despite these advances, the causal relationships between CO poisoning and inflammatory cytokines remain unclear. It is uncertain whether inflammatory cytokines predispose individuals to CO toxicity or if CO exposure subsequently alters cytokine expression. To address these questions, we applied a bidirectional 2-sample Mendelian randomization (MR) approach. The MR is an innovative method and the advantage of the MR approach is to adopt genetic variants as IVs, which effectively mitigates confounding factors and reverse causation.^[10]^ By investigating both directions – cytokines influencing CO toxicity risk and CO toxicity influencing cytokine levels – this study aims to clarify the interplay between inflammation and CO poisoning, potentially revealing novel therapeutic targets.

## 2. Materials and methods

### 2.1. Study design

**Figure 1.**
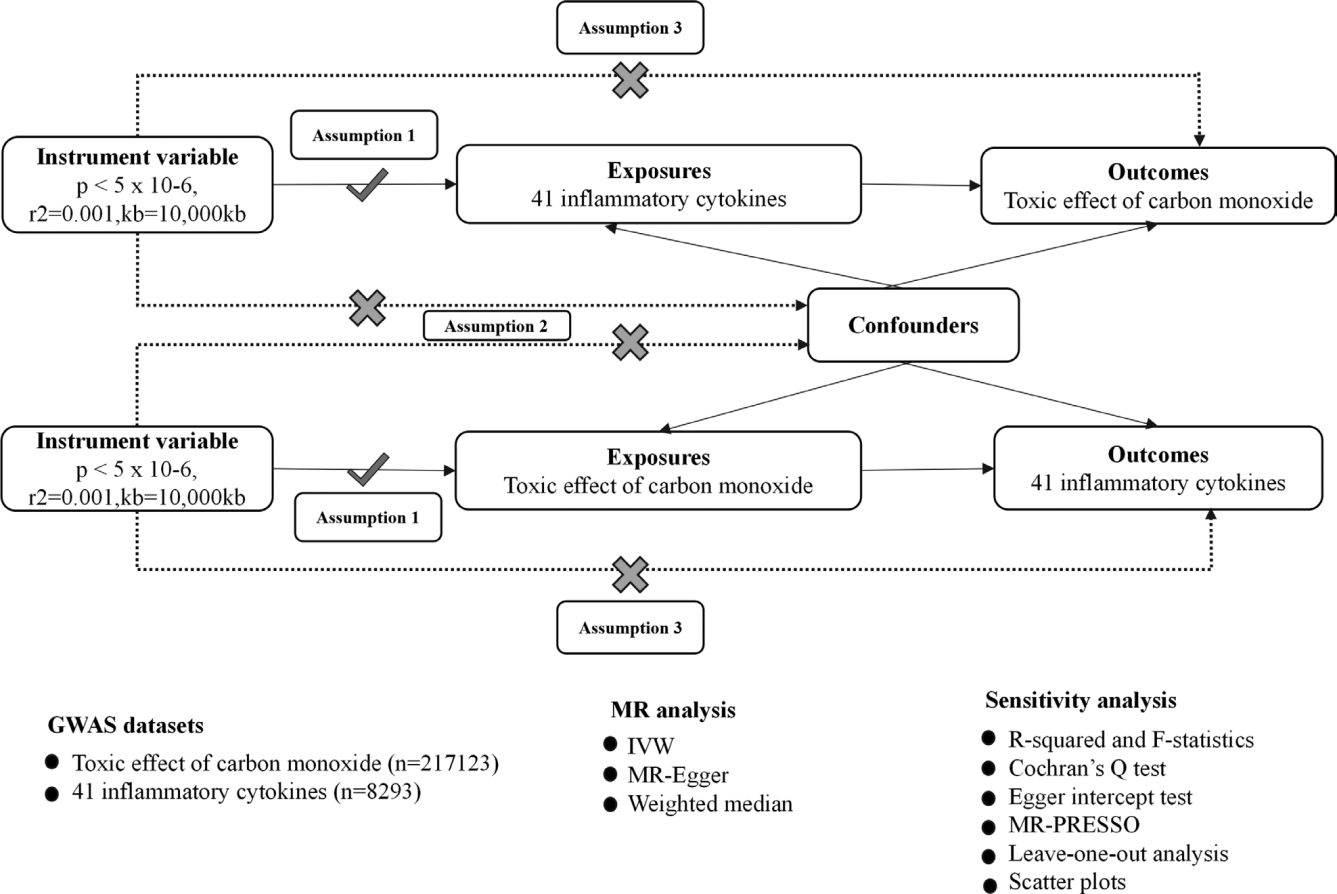
Overview of the MR design. GWAS = genome-wide association study, IVW = inverse-variance weighted, MR = Mendelian randomization, MR-PRESSO = Mendelian Randomization Pleiotropy RESidual Sum and Outlier.

A 2-sample bidirectional MR analysis was implemented through 2 distinct datasets representing the exposure and outcome, so as to examine the potential causation between CO toxicity and inflammatory cytokines. To attain unbiased estimations of MR, we followed 3 basic hypotheses.^[11]^ First, the initial assumption asserts that genetic variants as instruments should show a robust connection with the exposure. The second hypothesis entails that the variants are still unaffected by any confounding variables that may influence the outcome. The last hypothesis indicates that genetic instruments are supposed to impact the outcome thoroughly via the exposure. Figure 1 describes the flow chart for MR design.

### 2.2. Data sources

In the MR analysis, 2 datasets were sourced from openly available genome-wide association study (GWAS) summary statistics (Additional file: Table S1, Supplemental Digital Content, https://links.lww.com/MD/P983). Specifically, genetic instruments pertaining to inflammatory cytokines originated in the widest comprehensive GWAS conducted on inflammatory cytokines, which included genotyping information from 8293 Finnish persons throughout 3 discrepant cohorts: the Cardiovascular Risk in Young Finns Study, FINRISK1997, and FINRISK2002. This dataset is available for public access at the website: https://doi.org/10.5523/bris.3g3i5smgghp0s2uvm1doflkx9x.

The summary statistics of the toxic effect of CO included 231 cases and 2,16,892 controls of European ancestry. The GWAS data is capable of being publicly assessed via the IEU Open GWAS project. The corresponding link is: https://gwas.mrcieu.ac.uk/.

Based on the origin of the cohorts, there is no sample overlap between the cytokine GWAS and the toxic effect of CO GWAS.

### 2.3. Instrumental variables selection

Initially, single nucleotide ploymorphisms (SNPs) linked to the exposure were collected using the GWAS significance criteria, applying a slightly relaxed threshold of *P* < 5E−06 for inflammatory cytokines and CO toxicity (Additional file: Tables S2 and S3, Supplemental Digital Content, https://links.lww.com/MD/P983). This adjustment was necessary, as only a limited number of SNPs were identified when a more strict threshold of *P* < 5E−08 was utilized for both parameters. To obtain independent IVs, a clumping procedure was executed according to the traditional linkage disequilibrium threshold, specifically *r*² < 0.001 showing a minimal distance of 10,000 kb. This approach utilized the linkage disequilibrium reference panel from the 1000 Genomes Project. Following the harmonization process, we eliminated palindromic and ambiguous SNPs. At last, to rate the credibility of the IVs, the *R*-squared (*R*²) and *F*-statistics were figured out. An *F*-statistic value over 10 was regarded as a mark of a strong instrument.^[12]^ The calculations were performed through the equations as follows: *R*² = (2β² × MAF × (1 − MAF))/(2β² × MAF × (1 − MAF) + 2N × MAF × (1 − MAF) × SE²), *F* = ((N − 2) × (*R*²/(1 − *R*²))). In these formulas, MAF refers to the minor allele frequency, β denotes the effect estimation of the SNP in the GWAS for exposure, and N indicates the sample size for the exposure.

### 2.4. MR analysis

The random-effects inverse-variance weighted (IVW) MR was utilized as the principal analytical approach.^[13]^ As part of our sensitivity analyses, MR-Egger^[14]^ and weighted median-based^[15]^ methods were executed to rate the robustness of the IVW MR findings. Furthermore, the Egger intercept test was adopted to account for any potential effect of horizontal pleiotropy regarding the genetic instruments.^[16]^ To evaluate heterogeneity in the selected IVs, Cochran's *Q* test (*P* < .05) was implemented.^[17]^ Additionally, we employed the Mendelian Randomization Pleiotropy RESidual Sum and Outlier (MR-PRESSO) approach, a robust method designed to identify horizontal pleiotropy and outliers through the "MR-PRESSO" package.^[18]^

The IVW estimations were regarded as an indicator of causalities just when they demonstrated congruence in statistical significance and direction with not less than 1 sensitivity analysis, while also lacking the pleiotropy evidence (*P* > .05). The significance threshold was *P* < .05. All analyses were conducted in R software (version 4.4.1) using the TwoSampleMR, MRInstruments, data.table, vroom, plyr, dplyr and ggplot2 packages.

## 3. Results

### 3.1. The causal effect of inflammatory cytokines on the toxic effect of CO

**Figure 2.**
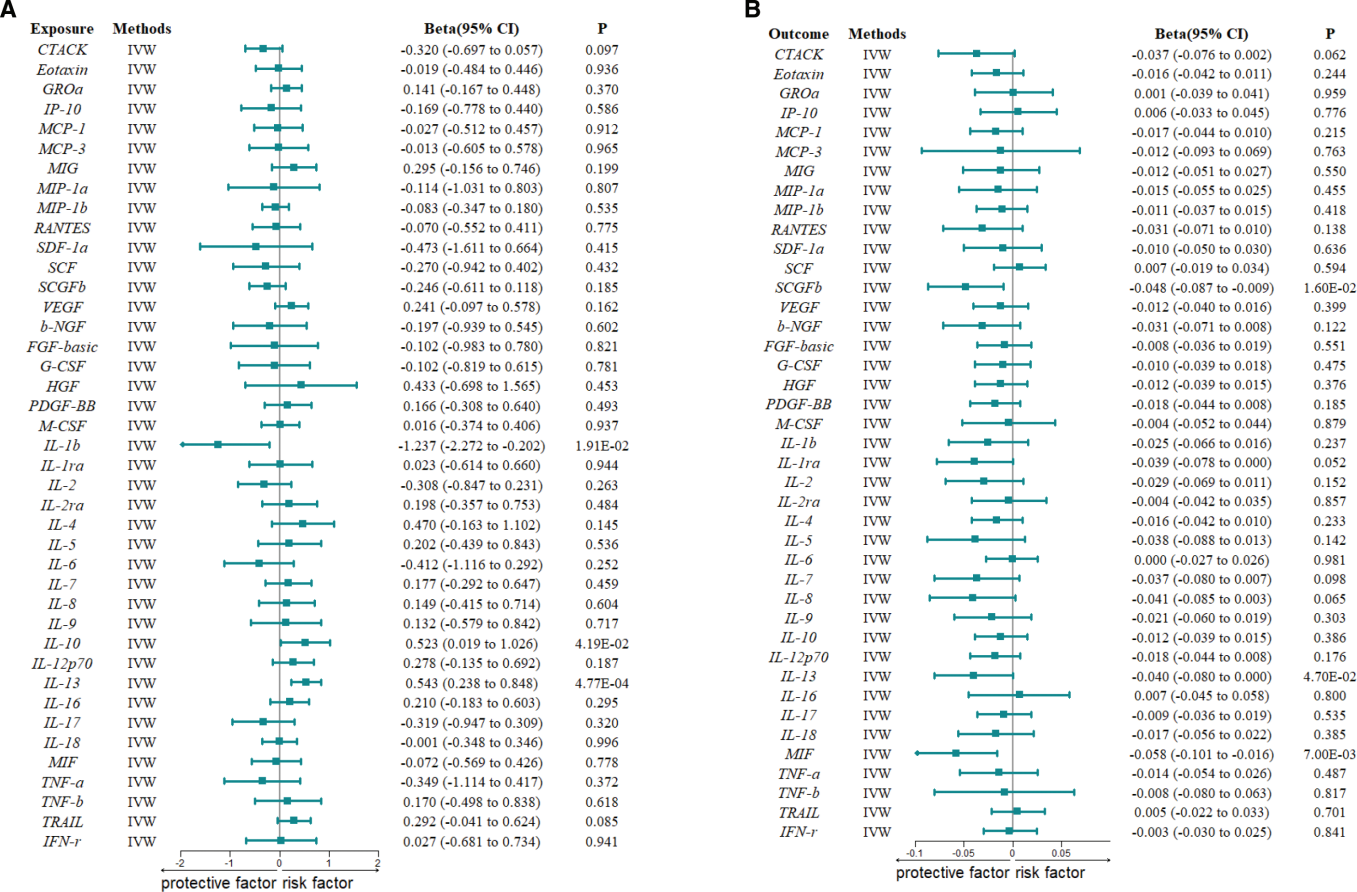
Inverse-variance weighted (IVW) Mendelian randomization (MR) estimates for the causal effects between inflammatory cytokines and toxic effect of carbon monoxide (CO). The vertical dashed line indicates the null value (β = 0). β > 0 suggests a positive causal effect, while β < 0 indicates a potential protective effect. (A) Forward MR analysis: causal effect of inflammatory cytokines on CO toxicity. (B) Reverse MR analysis: causal effect of CO toxicity on inflammatory cytokines. CI = confidence interval, CO = carbon monoxide, IVW = inverse-variance weighted, MR = Mendelian randomization.

**Figure 3.**
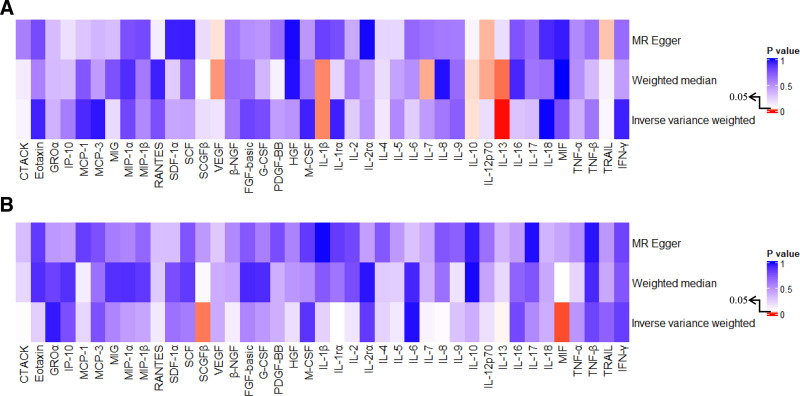
Heatmap of Mendelian randomization (MR) estimates for the causal effects between 41 inflammatory cytokines and toxic effect of carbon monoxide (CO). (A) Forward MR analysis: causal effect of inflammatory cytokines on CO toxicity. (B) Reverse MR analysis: causal effect of CO toxicity on inflammatory cytokines. CO = carbon monoxide, MR = Mendelian randomization.

Our bidirectional 2-sample MR analysis revealed that genetically predicted lower levels of IL-1β and higher levels of IL-10 and IL-13 were associated with an increased risk of CO toxicity. Specifically, the inverse variance weighted (IVW) estimates indicated a protective effect of IL-1β (β = −1.23; 95% CI: −2.27, −0.20; *P* = 1.91 × 10⁻^2^), while both IL-10 (β = 0.52; 95% CI: 0.02, 1.03; *P* = 4.19 × 10⁻^2^) and IL-13 (β = 0.54; 95% CI: 0.24, 0.85; *P* = 4.77 × 10⁻^4^) appeared to contribute to elevated susceptibility to CO poisoning. These β coefficients represent the change in genetically predicted CO toxicity risk per 1 − standard deviation increase in cytokine concentration. The findings are visualized in Figure 2A (forest plot of IVW estimates) and Figure 3A (heatmap of 3 MR methods), with detailed numerical results presented in Tables S4 to S6, Supplemental Digital Content, https://links.lww.com/MD/P983.

Results were directionally consistent when applying the weighted median method, suggesting robustness to potential invalid instruments. While MR-Egger regression did not yield statistically significant associations, the effect directions were aligned with the IVW estimates, enhancing the credibility of the causal inference (Table 1, Fig. 3A). In accordance with our prespecified MR criteria, IVW estimates were interpreted as indicative of causal relationships only when they showed consistent direction and statistical significance with at least 1 sensitivity analysis (weighted median or MR-Egger), and when no evidence of directional pleiotropy was detected. These criteria were met for IL-1β, IL-10, and IL-13 (see Table 1 and Fig. 3A), which are therefore considered to have robust evidence of causal association with CO toxicity. In Figure 3A, red cells indicate *P* < .05, while blue cells indicate non-significance, enabling visual comparison across methods.

**Table 1:** Sensitivity analysis for the causal effect of IL-1β, IL-10, and IL-13 on toxic effect of CO.

Exposure	Weighted median		MR-Egger regression		Heterogeneity	MR-PRESSO outlier detect		Pleiotropy
	β (95% CI)	*P* value	β (95% CI)	*P* value		β (95% CI)	*P* value	
IL-1β	−1.543 (−2.837, −0.249)	.019	−0.362 (−2.960, 2.236)	.830	Cochrane *Q* = 0; *P* = .557	NA		Intercept = −0.232; *P* = .603
IL−10	0.647 (0.027, 0.167)	.041	1.006 (−0.086, 2.099)	.096	Cochrane *Q* = 15; *P* = .258	No significant outliers		Intercept = −0.070; *P* = .347
IL−13	0.506 (0.103, 0.909)	.014	0.424 (−0.167, 1.014)	.187	Cochrane *Q* = 7; *P* = .759	No significant outliers		Intercept = 0.038; *P* = .653

CI = confidence interval, CO = carbon monoxide, IL = interleukin, MR = Mendelian randomization, MR-PRESSO = Mendelian Randomization Pleiotropy RESidual Sum and Outlier.

To assess the presence of horizontal pleiotropy and evaluate the robustness of the causal estimates, MR-PRESSO analysis was conducted for all 41 inflammatory cytokines. As shown in Table S6, Supplemental Digital Content, https://links.lww.com/MD/P983, MR-PRESSO did not identify any significant outliers for the majority of exposures, including IL-1β, IL-10, and IL-13, showing significant causal associations with CO toxicity in the primary MR analysis. Notably, IL-13 exhibited a highly significant raw *P*-value (*P* = 9.25 × 10⁻⁴), further supporting its potential causal role, yet no outliers were detected, reinforcing the validity of the IVW results.

**Figure 4.**
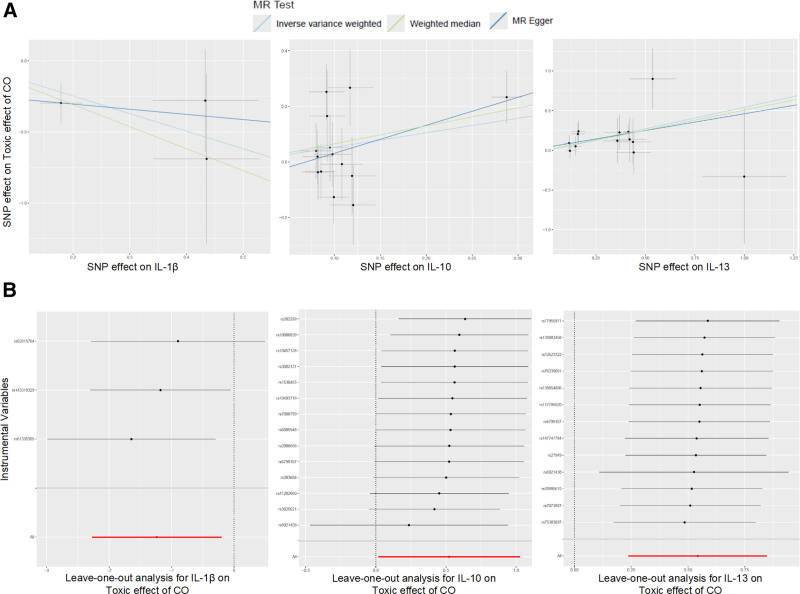
Scatter plots (A) and leave-one-out plots (B) for the causal effect of IL-1β, IL-10, and IL-13 on toxic effect of CO. CO = carbon monoxide, IL = interleukin, SNP = single nucleotide polymorphism.

No evidence of heterogeneity or horizontal pleiotropy was observed. Cochran's Q statistics yielded non-significant heterogeneity across SNPs for IL-1β, IL-10, and IL-13 (all *Q*_*P*val > 0.05), and MR-Egger intercept tests were also non-significant (*P* > .05), supporting the validity of the IVs (Table 1). The leave-one-out and scatter plots further confirmed the stability and consistency of these findings (Fig. 4A, B), ruling out undue influence from any single SNP. The scatter plots (Fig. 4A) display the SNP-specific causal estimates with standard errors, overlaid with regression lines from the IVW, weighted median, and MR-Egger methods. The alignment of these regression lines with the overall direction of effect reinforces the consistency and reliability of the MR findings. In the leave-one-out plots (Fig. 4B), each point represents the IVW estimate recalculated after removing 1 single nucleotide polymorphism (SNP) at a time; the consistent direction and magnitude across iterations indicate that no single SNP disproportionately influenced the overall association.

### 3.2. The causal effect of CO toxicity on inflammatory cytokines

In the reverse direction, using 10 SNPs associated with CO toxicity as IVs (Table S3, Supplemental Digital Content, https://links.lww.com/MD/P983), IVW MR analysis identified statistically significant inverse associations of CO toxicity with stem cell growth factor β (β = −0.05; 95% CI: −0.09, −0.01; *P* = 1.62 × 10⁻^2^), IL-13 (β = −0.04; 95% CI: −0.08, −0.001; *P* = 4.73 × 10⁻^2^), and macrophage migration inhibitory factor (β = −0.06; 95% CI: −0.10, −0.02; *P* = 7.42 × 10⁻^3^; Figs. 2B and 3B; Tables S7–S9, Supplemental Digital Content, https://links.lww.com/MD/P983).

To assess potential horizontal pleiotropy in the reverse MR analysis, MR-PRESSO was performed for all 41 inflammatory cytokines using the toxic effect of CO as the exposure. As shown in Table S9, Supplemental Digital Content, https://links.lww.com/MD/P983, no significant outliers were detected for any of the tested cytokines, suggesting that the causal estimates were not substantially influenced by pleiotropic IVs.

However, these associations did not withstand further scrutiny under sensitivity analyses. Both MR-Egger regression and the weighted median method failed to confirm the robustness of the IVW estimates (Table 2, Fig. 3B), indicating that the observed effects in the reverse direction should be interpreted with caution. Possible violations of IV assumptions or weak instrument bias may have contributed to these inconsistent results.

**Table 2:** Sensitivity analysis of the causal effect of toxic effect of CO on SCGFβ, IL-13, and MIF.

Outcome	Weighted median		MR-Egger regression		Heterogeneity	MR-PRESSO outlier detect		Pleiotropy
	β (95% CI)	*P* value	β (95% CI)	*P* value		β (95% CI)	*P* value	
SCGFβ	−0.045 (−0.096, 0.005)	.080	−0.044 (−0.154, 0.066)	.469	Cochrane *Q* = 3; *P* = .667	No significant outliers		Intercept = −0.002; *P* = .947
IL-13	−0.036 (−0.092, 0.020)	.209	−0.062 (−0.183, 0.059)	.358	Cochrane *Q* = 6; *P* = .334	No significant outliers		Intercept = −0.015; *P* = .716
MIF	−0.056 (−0.112, 0.001)	.053	−0.063 (−0.202, 0.075)	.419	Cochrane *Q* = 3; *P* = .505	No significant outliers		Intercept = 0.003; *P* = .941

CI = confidence interval, CO = carbon monoxide, IL = interleukin, MR = Mendelian randomization, MIF = macrophage migration inhibitory factor, MR-PRESSO = Mendelian Randomization Pleiotropy RESidual Sum and Outlier, SCGFβ = stem cell growth factor β.

## 4. Discussion

Our research put forward the first integrated investigation into the causality between inflammatory cytokines and the toxic effect of CO. The research finding was that a low level of IL-1β and a high level of IL-10 and IL-13 might intensify the hazard of CO poisoning. In reverse MR analysis, no significant causal effects of CO poisoning were observed across the 41 inflammatory cytokines (all *P* > .05).

IL-1β, a pro-inflammatory cytokine, plays a critical role in initiating inflammatory responses to tissue damage and infection. Emerging experimental evidence suggests that IL-1β serves as a crucial mediator in launching host defense mechanisms through neutrophil recruitment and innate immune system activation following tissue damage or sterile inflammatory challenges.^[19]^ In murine models, IL-1β deficiency or neutralization markedly impairs neutrophil infiltration and acute immune activation, leading to delayed tissue repair and worse outcomes in infection or ischemia model systems.^[20]^ Thus, genetically lower baseline IL-1β levels may reflect an impaired capability to mount effective early inflammatory responses against acute injury, including CO-induced hypoxic damage, which could predispose individuals to more severe CO poisoning.

Previous research has indicated that elevated levels of anti-inflammatory cytokines such as IL-10 and IL-13 typically confer protective effects in various inflammatory and infectious diseases by reducing tissue damage and promoting healing.^[21,22]^ However, their roles in acute toxic injuries such as CO poisoning may differ. In the context of CO poisoning, where a prompt and effective inflammatory response might be crucial for limiting initial damage, overexpression of IL-10 and IL-13 may paradoxically inhibit protective responses.

Although direct evidence is lacking for IL-10 levels predicting severity in CO poisoning, it is plausible that elevated IL-10, induced by CO exposure, may suppress critical early inflammatory responses needed to mitigate hypoxic and oxidative damage. Experimental studies indicate that CO exposure can strongly induce IL-10 expression via heme oxygenase-1 pathways, while simultaneously suppressing IL-1β secretion by inhibiting NLRP3 inflammasome activation and caspase-1 cleavage.^[23,24]^ IL-10 is also known to reduce reactive oxygen species (ROS) production and dampen NLRP3-dependent IL-1β maturation in microglia, thereby suppressing early pro-inflammatory signaling required for effective damage response (neuroinflammation models).^[25]^

IL-13, primarily secreted by T-helper type 2 (Th2) cells, is a multifunctional cytokine involved in immune regulation and inflammatory responses.^[26]^ Previous studies have demonstrated that IL-13 can exacerbate neuronal injury by promoting ROS accumulation and inducing mitochondrial dysfunction in various disease models. It is acknowledged that CO poisoning induces a hypoxic environment and raises the level of ROS which induces oxidative stress damage and acute neuronal damage. It is known that there exists a close relationship between IL-13 and ROS. For instance, studies have demonstrated that IL-13 exacerbates neurotoxicity induced by prothrombin Kringle-2 in the hippocampus, primarily through oxidative stress mechanisms.^[27]^ This indicates that IL-13 can potentiate the adverse impacts of ROS in the nervous system, leading to cellular damage and dysfunction. Reportedly, IL-4 and IL-13 induce the equivalent expressions of conventional M2 markers in human macrophages while modulating ROS levels, highlighting their role in macrophage polarization and inflammatory responses.^[28]^ This modulation of ROS by IL-13 could have implications for its involvement in chronic inflammations, in which oxidative stress contributes to tissue damage. Moreover, IL-13’s influence extends to mitochondrial function, where it can induce mitochondrial dysfunction and promote cellular senescence, as observed in IgG4-related sialadenitis.^[29]^ This mitochondrial dysfunction is often accompanied by increased ROS production, further exacerbating oxidative stress within cells. In the context of cardiovascular health, IL-13 has been shown to alleviate cardiomyocyte apoptosis via enhancing fatty acid oxidation in mitochondria, which may indirectly regulate ROS levels and protect against oxidative damage.^[30]^ The relationship between CO and ROS may explain our finding. Therefore, elevated IL-13 levels, triggered by CO exposure, may play a contributory role in the development of CO poisoning by intensifying oxidative stress, disrupting mitochondrial integrity, and altering key inflammatory pathways.

These findings collectively suggest that elevated IL-10 and IL-13 may predispose individuals to worse outcomes by inhibiting necessary innate immune responses and enhancing oxidative stress. While further clinical validation is warranted, current mechanistic data provide a biologically plausible foundation for our MR findings.

## 5. Limitation

### 5.1. Methodological assumptions: linearity, lifetime exposure and external validation

First, a major methodological limitation of MR is the assumption of a linear relationship between exposure and outcome. In toxicological settings, the dose-response relationship may be nonlinear, with potential threshold or saturation effects which may lead to underestimation or oversimplification of causal effects.^[31]^ Second, MR estimates the effects of genetically predicted lifelong exposure,^[32]^ whereas CO poisoning typically results from acute or short-term exposure. This mismatch in exposure timing may affect interpretation, particularly in the context of immediate inflammatory responses. As such, our study is more reflective of how chronic inflammatory states or long-term immune predisposition may affect susceptibility to CO toxicity, rather than modeling acute cytokine changes following exposure. Third, although our findings were derived from large-scale GWAS summary statistics and supported by multiple sensitivity analyses, no external replication was conducted using independent datasets.

These factors together highlight important methodological limitations. Future studies with access to individual-level data and larger sample sizes should apply non-linear MR methods^[33]^ and time-sensitive designs (e.g., acute or time-series models) to better capture these complex exposure–outcome dynamics and validate our findings.

### 5.2. Instrumental variables and pleiotropy

Although we evaluated the 3 core IV assumptions underlying MR, potential violations cannot be entirely excluded. The relevance assumption was supported by strong instruments (*F*-statistics > 10), while the independence and exclusion restriction assumptions were assessed using MR-Egger intercept, Cochran’s *Q* statistic, and MR-PRESSO. These tests suggested no evidence of directional pleiotropy or significant heterogeneity. However, undetected horizontal pleiotropy may persist, particularly given the biological complexity of inflammatory regulation.

### 5.3. Limited sample size and population generalizability

A key limitation of our study is the small number of CO poisoning cases (n = 231) in the outcome GWAS, which substantially constrained our statistical power - particularly for detecting potential reverse causal effects of CO toxicity on cytokine levels. While we employed rigorous MR methods and sensitivity analyses, this limited sample size increases the risk of both type II errors (false negatives) in our reverse MR analyses and imprecision in our effect estimates.^[34,35]^ Importantly, the Finnish/European ancestry of all included datasets further restricts the generalizability of our findings to other populations, where genetic architectures and environmental exposures may differ substantially. To address these limitations, we strongly recommend replication of these analyses in: significantly larger GWAS of CO poisoning to improve power, particularly for reverse causation testing; and diverse, multi-ethnic cohorts to evaluate the trans-ancestry robustness of these cytokine-CO toxicity relationships. Such validation efforts would substantially strengthen confidence in these findings and their potential clinical applicability across populations.

### 5.4. Incomplete mechanistic interpretation and unmeasured confounders

While this study examined 41 inflammatory cytokines, it is possible that other relevant inflammatory or metabolic pathways involved in CO poisoning were not captured, potentially affecting the comprehensiveness of the analysis. The complex pathophysiology of CO poisoning – encompassing oxidative stress, mitochondrial dysfunction, and hypoxia-induced damage – may only be partially explained by inflammatory signaling. Moreover, potential gene-environment interactions and subgroup-specific effects warrant further consideration. Although our MR approach minimizes confounding, it does not account for important effect modifiers such as sex, age, or environmental co-exposures like smoking. These factors are known to influence both cytokine expression and susceptibility to CO poisoning.^[35]^ For instance, smoking has been shown to alter baseline inflammatory profiles, including IL-1β and IL-10 levels,^[36]^ which may interact with genetic predisposition to affect CO-related outcomes. Similarly, age-related immune changes and sex-based hormonal influences could modulate cytokine responses to toxic exposures.^[37,38]^ Future studies incorporating stratified analyses or gene-environment interaction models are needed to fully elucidate how these variables may shape the cytokine–CO toxicity relationship.

## 6. Conclusions

In summary, this MR study provides genetic evidence supporting a potential causal role of IL-10, IL-1β, and IL-13 in the pathogenesis of CO poisoning. These findings offer mechanistic insights into how inflammatory cytokines may modulate susceptibility or response to CO toxicity, highlighting novel targets for potential therapeutic intervention.

However, the interpretation of these results must remain cautious, given the inherent assumptions of the MR approach and the limitations discussed above, including potential residual pleiotropy, limited phenotype coverage, and modest statistical power for the outcome GWAS.

While our findings are broadly consistent with existing experimental and observational studies implicating IL pathways in CO-induced inflammation and tissue injury, MR offers a complementary line of causal inference. Nonetheless, further investigations – particularly in larger, multi-ethnic cohorts and functional studies – are necessary to validate and expand upon these associations.

## Acknowledgments

We thank the studies and consortiums referenced and included in the present analysis for providing public datasets. We thank the editors and reviewers for their helpful suggestions that improved this article.

## Author contributions

**Conceptualization:** Huan Yang.

**Data curation:** Huan Yang.

**Formal analysis:** Huan Yang.

**Investigation:** Huan Yang.

**Methodology:** Huan Yang.

**Software:** Huan Yang.

**Supervision:** Manli Sun, Yulei Bi.

**Validation:** Lijie Zhao, Jingqi Ruan, Shichang Li, Yang Xu, Mingjing Duan.

**Writing – original draft:** Huan Yang.

## Supplementary Material


